# Anatomic risk factors for the occurrence of medial talar osteochondral lesions: a case–control study

**DOI:** 10.1007/s00256-022-04024-6

**Published:** 2022-03-24

**Authors:** Lena Sonnow, Tarek Omar Pacha, Maximilian Richter, Dilek Yapar, Mustafa Cetin, Omer Faruk Celik, Ozkan Kose

**Affiliations:** 1 Institute of Diagnostic and Interventional Radiology, Medical School Hannover, Carl-Neuberg-Straße 1, 30625 Hannover, Germany; 2 Department of Traumatology, Medical School Hannover, Hannover, Germany; 3 General Practice Center Neustadt, Hannover, Germany; 4 Department of Public Health, Ministry of Health Antalya Muratpasa District Health Directorate, Antalya, Turkey; 5 Department of Biostatistics and Medical Informatics, Faculty of Medicine, Akdeniz University, Antalya, Turkey; 6 Department of Radiology, Antalya Education and Research Hospital, Antalya, Turkey; 7 Department of Orthopedics and Traumatology, Antalya Education and Research Hospital, Antalya, Turkey; 8 Department of Anatomy, Faculty of Medicine, Akdeniz University, Antalya, Turkey

**Keywords:** Osteochondral lesions of the talus, Osteochondritis dissecans, Talus, Anatomy, Etiology, Risk factors

## Abstract

**Objective:**

This study aimed to determine the anatomical risk factors that may play a role in the etiology of medial-sided osteochondral lesions of the talus (OLT) using morphological parameters in magnetic resonance imaging (MRI).

**Subjects and methods:**

One hundred twenty-four patients with medial-sided OLT and age- and sex-matched 124 controls were included in this retrospective study. Two examiners conducted independent OLT classification and measurements of five MRI parameters: tibial axis-medial malleolus angle (TMM), the anterior opening angle of the talus (AOT), talus position (TalPos), the ratio of the distal tibial articular surface to the length of the trochlea tali arc (TAS/TAL), depth of the incisura fibularis (IncDep). Statistical analysis included intraclass correlation coefficients, independent *t*-tests, receiver-operating characteristic (ROC) analysis, area under the curve (AUC) calculation, and logistic regression analysis. A *p*-value < 0.05 was considered statistically significant.

**Results:**

TTM, AOT, TalPos, and TAL values were significantly higher and the TAS/TAL ratio was significantly lower in the case group than in the control group (*p* < 0.001). Cut-off and AUC values for TMM were 15.15° (AUC 0.763), AOT 13.05° (AUC 0.826), TalPos 0.75 mm (AUC 0.887), TAL 35.45 mm (AUC 0.642), and TAS/TAL ratio 0.82 (AUC 0.784), *p* < 0.001. Multivariate logistic regression analysis results were odds ratio (OR) = 6.1 for TMM ≥ 15.15°, OR = 8.9 for AOT ≥ 13.05°, OR = 36.1 for TalPos ≥ 0.75 mm, and OR = 6.7 for TAS/TAL ratio ≤ 0.82.

**Conclusion:**

Ankle morphology might have an influence on OLT development. The talus position (TalPos) and anterior opening angle of the talus (AOT) seemed to be the strongest predisposing factors.

## Introduction

Osteochondral lesions of the talus (OLT) can be described as focal alterations of subchondral bone and cartilage with the risk of disruption of osteochondral fragments. This may lead to pain, swelling, mechanical symptoms, and premature ankle joint osteoarthritis in the long term [1, 2]. OLT might involve any location of the talar dome; however, medial-sided lesions are more common than other locations. Diepen et al. reviewed 2087 OLT cases within fifty-one studies in a recent meta-analysis. In this review, 73% of the cases were detected on the medial side (31% central-medial, 28% posteromedial, and 10% anteromedial), followed by lateral-sided lesions (24%) and central lesions (3%) [3].

Trauma has been proposed as the principal etiologic factor for lateral-sided OLT [4]. According to the recent literature, approximately 45% of ankle fractures are accompanied by lateral-sided OLT [5]. Although the relationship between lateral lesions and trauma has been widely accepted, the etiology of medial-sided lesions is not fully understood. Different hypotheses have been postulated, including vascular pathologies, systemic diseases, and genetic predisposition [1, 6–9].

Besides these theories, few authors reported that anatomic variations might be responsible for the occurrence of OLT [6, 10–12]. These authors argue that certain morphological traits create micro-instability, which deteriorates the articular contact pressures and consequently causes OLT by the cumulative effect of repetitive microtraumas. Previously, only a few anatomical parameters were studied on a limited number of patients. In the current study, new anatomical features that might be a predisposing factor to OLT were evaluated using magnetic resonance imaging (MRI).

## Subjects and methods

### Patients and study design

A retrospective chart review was done on all ankle MRI examinations performed in our institution between January 2018 and December 2020. A total of 1466 patients were detected who had ankle MRIs due to any reason, representing a heterogeneous population composed of acute trauma and emergency room cases, as well as outpatient clinics in a multi-specialty hospital setting. Among these patients, those with medial-sided OLT without a history of trauma were identified and evaluated for inclusion. Not only the MRI reports but also the MR images were reviewed by the radiologists; thus, the diagnosis was confirmed before inclusion in the study. Patients with concomitant alterations of ankle anatomy, such as tarsal coalitions, severe ligamentous lesions, and any other bony pathologies, were excluded. Another exclusion criterion was severe degeneration with a definite deformity of bone contour and large osteophytes, corresponding to a grade IV Kellgren-Lawrence grading system for osteoarthritis in plain radiographs. MRI-based diagnoses like soft tissue impingement, os trigonum syndrome, mild tendinopathies were not considered to be associated with significant bony anatomy changes and included in the evaluation. One hundred twenty-four patients (77 female/47 male) who met the inclusion/exclusion criteria were selected and included in the case group. One hundred twenty-four age- and sex-matched control patients without OLT were randomly selected and included in the study using the same data. Similar inclusion and exclusion criteria were applied for the control cases. The mean age difference between the case and control groups was 1.7 ± 0.9 years (range, 0–5 years). The demographic characteristics of the patients are presented in Table 1. The study was carried out in accordance with the ethical standards laid down in the 1964 Declaration of Helsinki and its later amendments. The institutional review board approved the study protocol (IRB approval number: 2021/14.6–289).

**Table 1:** Demographic characteristics of the patients in case and control groups

Variables		Case group *n* = 124	Control group *n* = 124	*p*-value
Sex				
Male, *n* (%)		47 (37.9%)	47 (37.9%)	1.000*
Female, *n* (%)		77 (62.1%)	77 (62.1%)	
Age (years)				
Total	Mean ± SD (range)	46.8 ± 13.7 (12–78)	45.5 ± 13.6 (13–77)	0.437**
Male	Mean ± SD (range)	44 ± 13.8 (13–78)	45.7 ± 15.3 (14–77)	0.568**
Female	Mean ± SD (range)	48.5 ± 13.5 (12–66)	45.3 ± 12.6 (13–74)	0.127**

^*^Pearson’s chi-square test; **independent samples *T*-test

### Image acquisition and post-processing

MRI scans were performed on a 1.5 T MRI Achieva DS Advance (Philips Healthcare, Eindhoven, The Netherlands) using an 8-channel ankle coil with the ankle joint in a neutral position. Sequences followed a standard ankle protocol from clinical routine comprising proton density–weighted (PDw) spectral presaturation inversion recovery (SPIR) sequences, a T1w turbo spin-echo (TSE) sequence, and a T2w SPIR sequence. All images were transferred to a client–server-based picture archiving and communication system (PACS) digital workstation (Sectra IDS7, Ver. 18.2., Sectra AB).

### MRI assessment and measurements

Based on previous studies, five parameters that represent ankle morphometry were determined [13, 14]. These parameters were specifically chosen because they were assumed to be a predisposing factor for ankle instability or increased articular contact pressures.

**Fig. 1:**
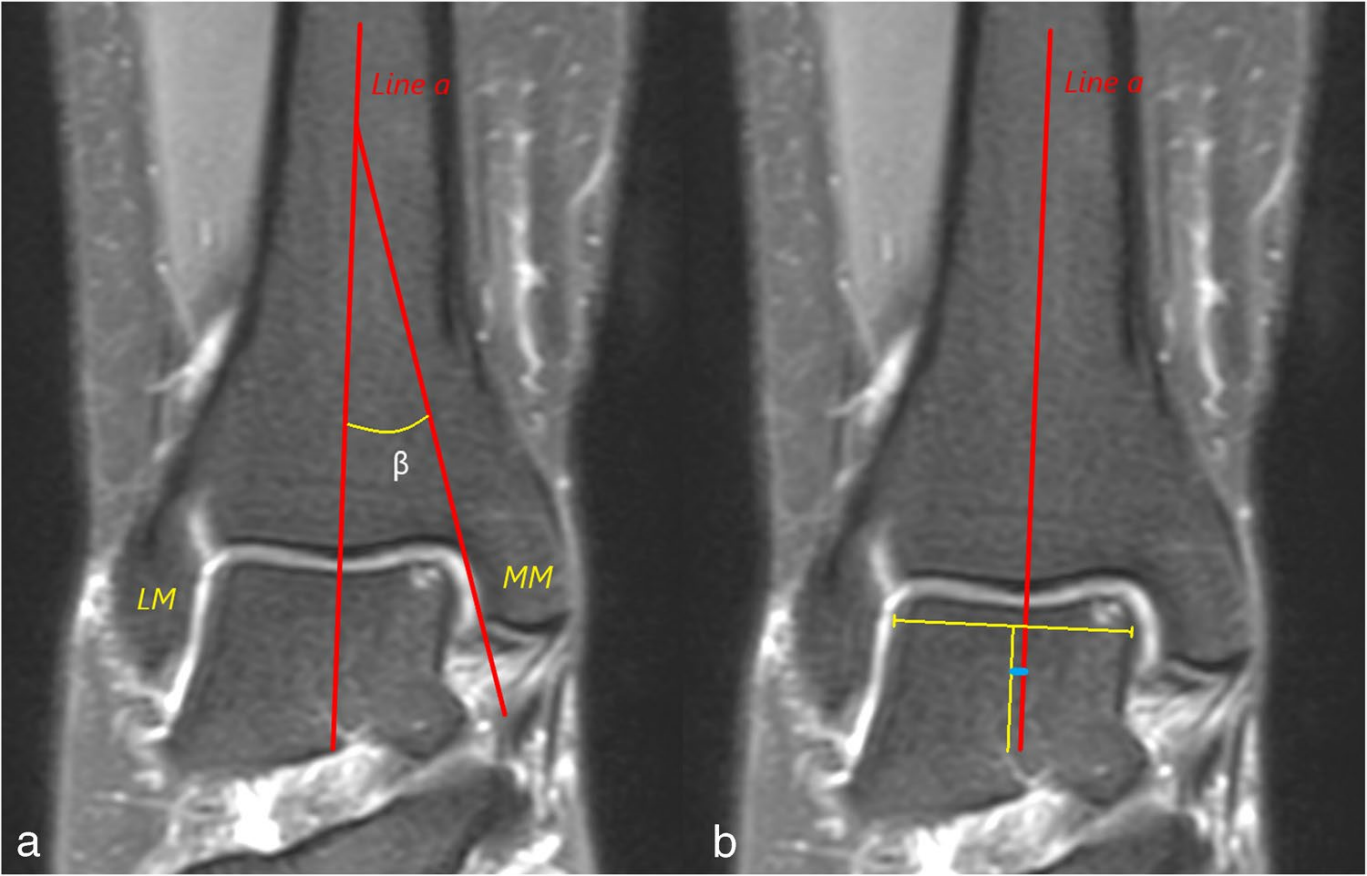
(a) The tibial axis-medial malleolus (TMM) angle measurement. Line a is the longitudinal axis of the tibial shaft. TMM is the angle (β) between line a and a line parallel to the medial malleolar joint surface. (b) The measurement of talus position (TalPos). Line a is the longitudinal axis of the tibial shaft. The perpendicular distance between Line a and the center of the talus is the deviation of the talus position within the mortise. Both measurements are performed using PDw coronal images

**Fig. 2:**
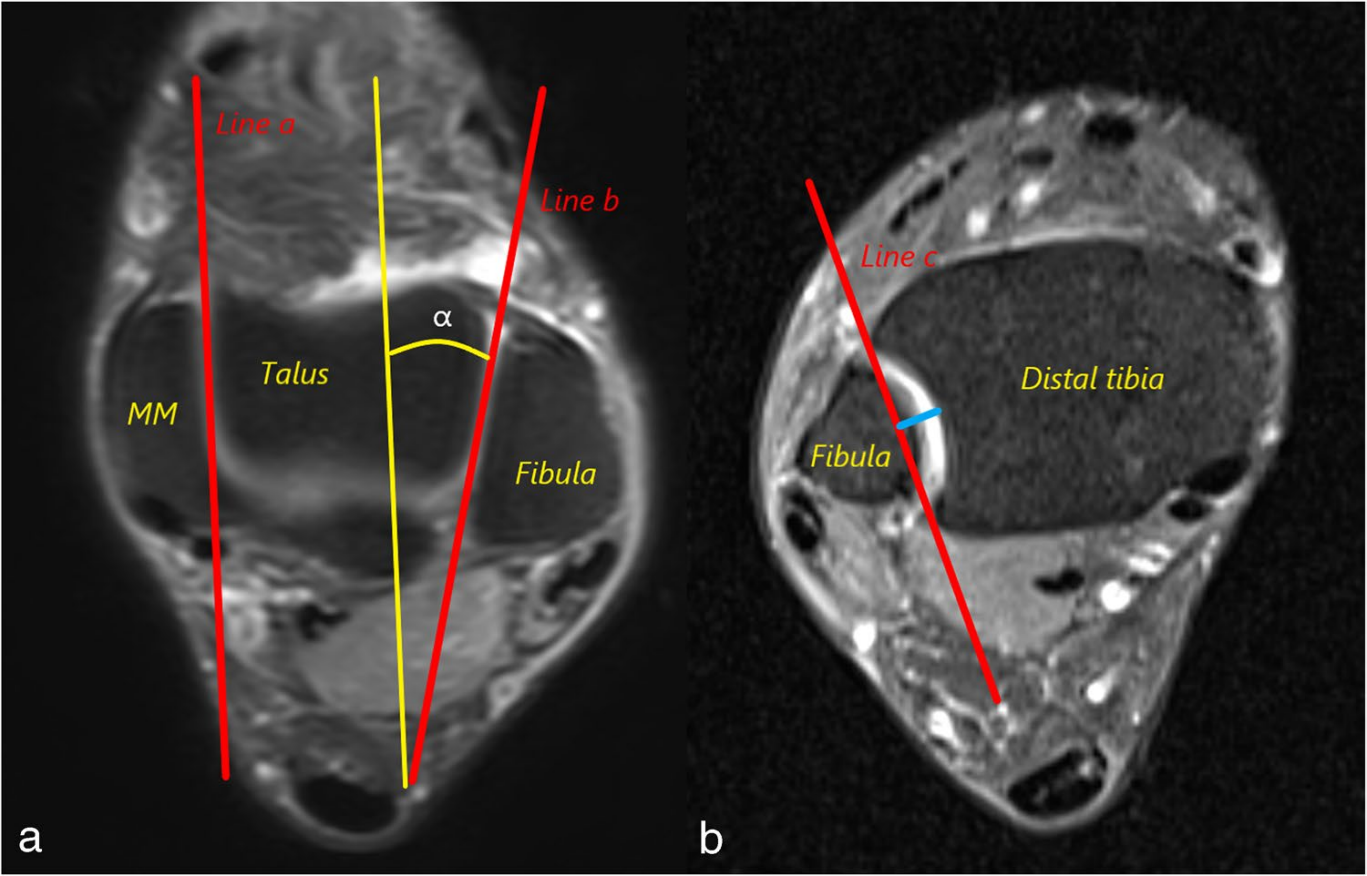
(a) Measurement of the anterior opening angle of the talus (AOT). Two lines are drawn that pass through the medial (Line a) and lateral (Line b) surfaces of the talar trochlea. The angle (α) between these lines is measured as AOT. (b) The measurement of incisura fibularis depth (IncDep). Line c is drawn between the anterior and posterior margins of the incisura fibularis. The perpendicular distance between Line c and the deepest point of the incisura is measured as IncDep (blue line)

**Fig. 3:**
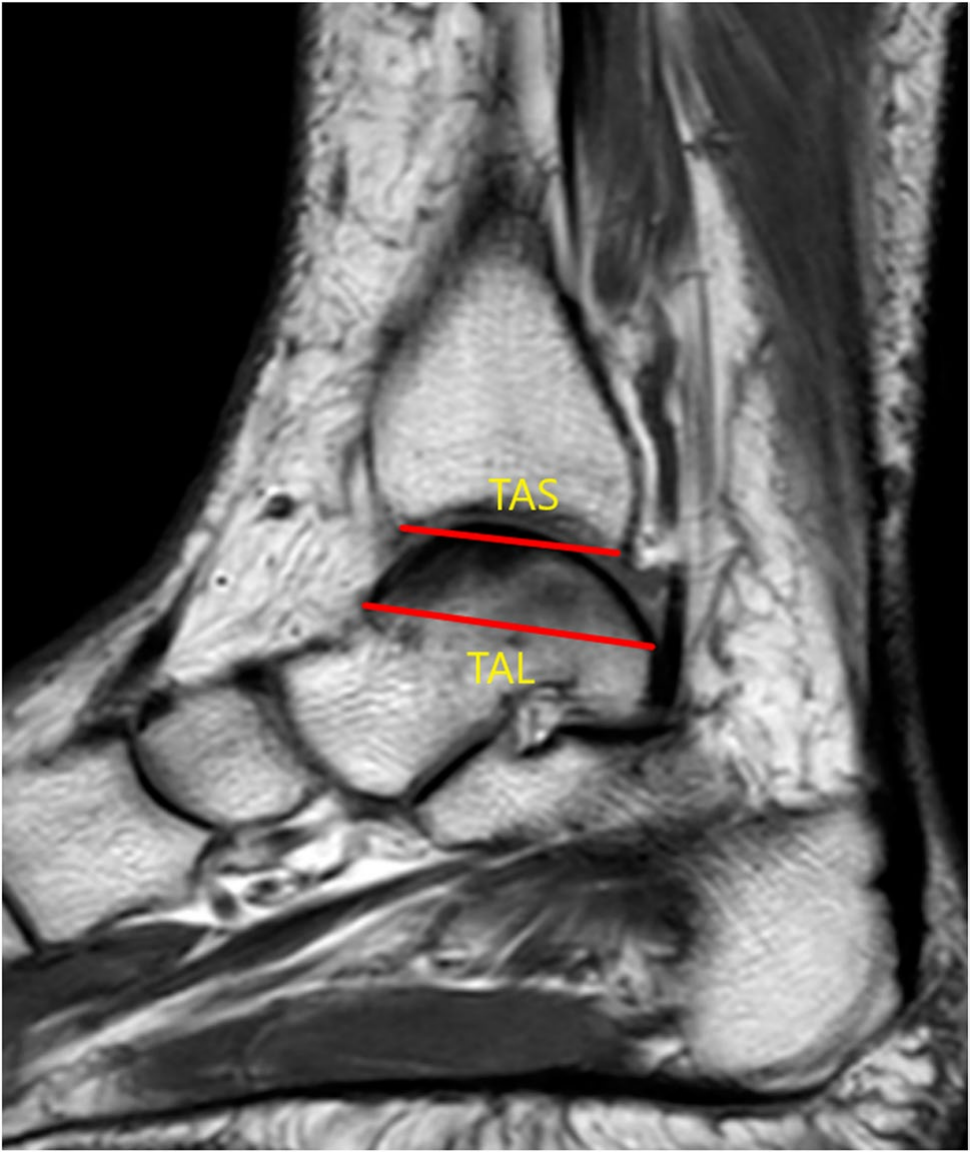
The measurement of the distal tibial articular surface (TAS) and the length of the trochlea tali arc (TAL)

The tibial axis-medial malleolus (TMM) angle and the talus position (TalPos) were evaluated on the coronal PDw images. The TMM angle is the angle between the tibial shaft and the joint surface of the medial malleolus (Fig. 1a). Sugimoto et al. have shown that the TMM angle on an anteroposterior radiograph is larger in patients with more severe talar chondral lesions [15]. The talus position (TalPos) is determined as the distance between the tibial shaft and the central point of the talus on the coronal plane. Positive values were considered a lateral deviation of the talus center and negative values as a medial deviation of the talus center in reference to the tibial axis (Fig. 1b). It is well-known that tibiotalar articular contact pressure is closely related to the talar position within the mortise [16]. Axial PDw images served for measuring the anterior opening angle of the talus (AOT) and the depth of the incisura fibularis. The AOT was recorded as the angle between the medial and lateral surfaces of the talar trochlea (Fig. 2a). The incisura fibularis (IncDep) depth was measured at the level of the distal tibiofibular syndesmosis as the distance between a tangent line to the prominent anterior and posterior margins and the deepest point of the incisura fibularis (Fig. 2b). Shallow fibular incisura has been shown as a risk factor for syndesmotic injury [17]. The maximal sagittal extension of the distal tibial articular surface (TAS) and the length of the trochlea tali arc (TAL) were determined on sagittal T1w images. The TAS was measured as the distance between the most anterior and posterior points of the distal tibial articular surface. The TAL was obtained by the distance from the most anterior and posterior points of the trochlea tali (Fig. 3). For the purpose of normalization, the TAS/TAL ratio was calculated by dividing the maximal tibial articular surface (TAS) by the length of the trochlea tali arc (TAL). It was assumed that as the surface area of the distal tibia decreases, the articular contact pressure would increase.

One experienced musculoskeletal radiologist (LS) and an orthopedic surgeon (TP) evaluated all MR images and performed the measurements on the digital workstation. Each observer performed radiologic assessments in random order on two separate occasions, at least 3 weeks apart. Observers were blinded to their and the other observer’s ratings.

### Statistical analysis

The statistical analyses were performed using the SPSS Statistics Base v. 23 software, and a *p*-value less than 0.05 was considered statistically significant. The categorical variables were presented as counts (*n*) and percentages (%), and continuous variables were presented as mean ± standard deviation (SD), median, and range for descriptive analyses. The normality of the data was assessed with visual histograms and probability graphics and tested using the Kolmogorov–Smirnov test. For the data that did not fit the normal distribution, the Mann–Whitney *U* test was used to compare the two independent groups, and the independent sample *t*-test was used for the data that fit the normal distribution. The chi-square test was used to compare categorical variables between separate groups. Kappa values for categorical variables and interclass correlation coefficient (ICC) values for continuous variables were calculated to assess intra-observer and inter-observer agreement.

Receiver-operating characteristic (ROC) analysis was used to determine optimal cut-off points of TMM, TalPos, TAL, and TAS/TAL ratio to predict the presence of an OLT. The area under the ROC curve (AUC) was considered excellent for AUC values between 0.9 and 1, good for AUC values between 0.8 and 0.9, fair for AUC values between 0.7 and 0.8, poor for AUC values between 0.6 and 0.7, and failed for AUC values between 0.5 and 0.6 [18, 19]. In this study, optimal cut-off points were decided by using 3 approaches together; (1) having the maximum specificity and sensitivity values, and (2) a positive LHR value of 2 and above, and (3) the values with the maximum Youden indexes were selected as the optimal cut-off values. Youden’s index was calculated as maximum (sensitivity + specificity − 1)] [20].

Variables (TMM, AOT, TalPOS, TAL, and TAS/TAL ratio) that may be independent association factors for OLT were evaluated by multivariate logistic regression analysis. Variables with *P* < 0.05 in the univariate analysis were entered into multivariate logistic regression analysis. The classic Bonferroni correction was used to control the familywise errors for multiple logistic regression models. Assuming seven predictors for OLT, *p* values < 0.007 were considered significant in this analysis (*α*/number of predictors = 0.05/7 = 0.007).

## Results

### Post hoc power analysis

We ran a post hoc power analysis, using G*Power Version 3.0.10 for a total sample size of 248 participants, allocation ratio = 1, *α* of 5%, and an effect size of 1.08 resulting from the difference between two independent means (TAS/TAL ratio) and found that this sample would have achieved 100% power.

### OLT staging

According to the OLT staging results, most of the lesions contained a subchondral cyst and were classified stage 5 by both readers (reader A1: 44 lesions, 17.7%; reader B1: 53 lesions, 21.4%). The second most lesion stage was 2a comprising cartilage injury with bony fracture and edema (reader A1: 34 lesions, 13.7%; reader B1: 33 lesions, 13.3%). The inter-observer reliability between both readers was excellent (kappa value = 0.929, *p* < 0.001).

### Evaluation of intra- and inter-observer agreement for anatomical measurements

The intra-observer and inter-observer consistency was evaluated by *interclass correlation coefficient* analysis for the anatomical measurements. All ICC values were found to be > 0.876 (range; 0.876–0.997) (Table 2), suggesting an excellent correlation.

**Table 2:** Evaluation of intra-observer and inter-observer agreement for measurements*.* Abbreviations, *A* observer A, *B* observer B, *t*_*1*_ first time, *t*_*2*_ second time, *TMM* tibial axis-medial malleolus angle, *AOT* anterior opening angle of the talus, *TalPos* talus position, *TAS* tibial articular surface, *TAL* length of trochlea tali arc, *IncDep* incisura depth, *ICC* intraclass correlation coefficient, *CI* confidence interval. *p* < 0.001 for all comparisons

Anatomical parameters	Intra-observer reliability ICC (95% CI)		Inter-observer reliability ICC (95% CI)	
	*A t*_*1*_* vs. A t*_*2*_	*B t*_*1*_* vs. B t*_*2*_	*A t*_*1*_* vs. B t*_*1*_	*A t*_*2*_* vs. B t*_*2*_
TMM	0.976 (0.969–0.981)	0.981 (0.976–0.985)	0.992 (0.990–0.994)	0.967 (0.958–0.975)
AOT	0.997 (0.996–0.997)	0.993 (0.991–0.995)	0.996 (0.995–0.997)	0.996 (0.995–0.997)
TalPos	0.997 (0.996–0.997)	0.996 (0.995–0.997)	0.997 (0.997–0.998)	0.997 (0.996–0.998)
TAS	0.984 (0.980–0.988)	0.953 (0.940–0.964)	0.971(0.963–0.978)	0.968 (0.959–0.975)
TAL	0.990 (0.987–0.992)	0.976 (0.969–0.981)	0.987 (0.983–0.990)	0.983 (0.987–0.987)
TAS/TAL ratio	0.945 (0.929–0.957)	0.878 (0.843–0.905)	0.945 (0.929–0.957)	0.876 (0.841–0.904)
IncDep	0.992 (0.990–0.994)	0.984 (0.979–0.988)	0.992 (0.989–0.994)	0.988 (0.985–0.991)

### Evaluation of anatomical measurements between case and control groups

**Fig. 4:**
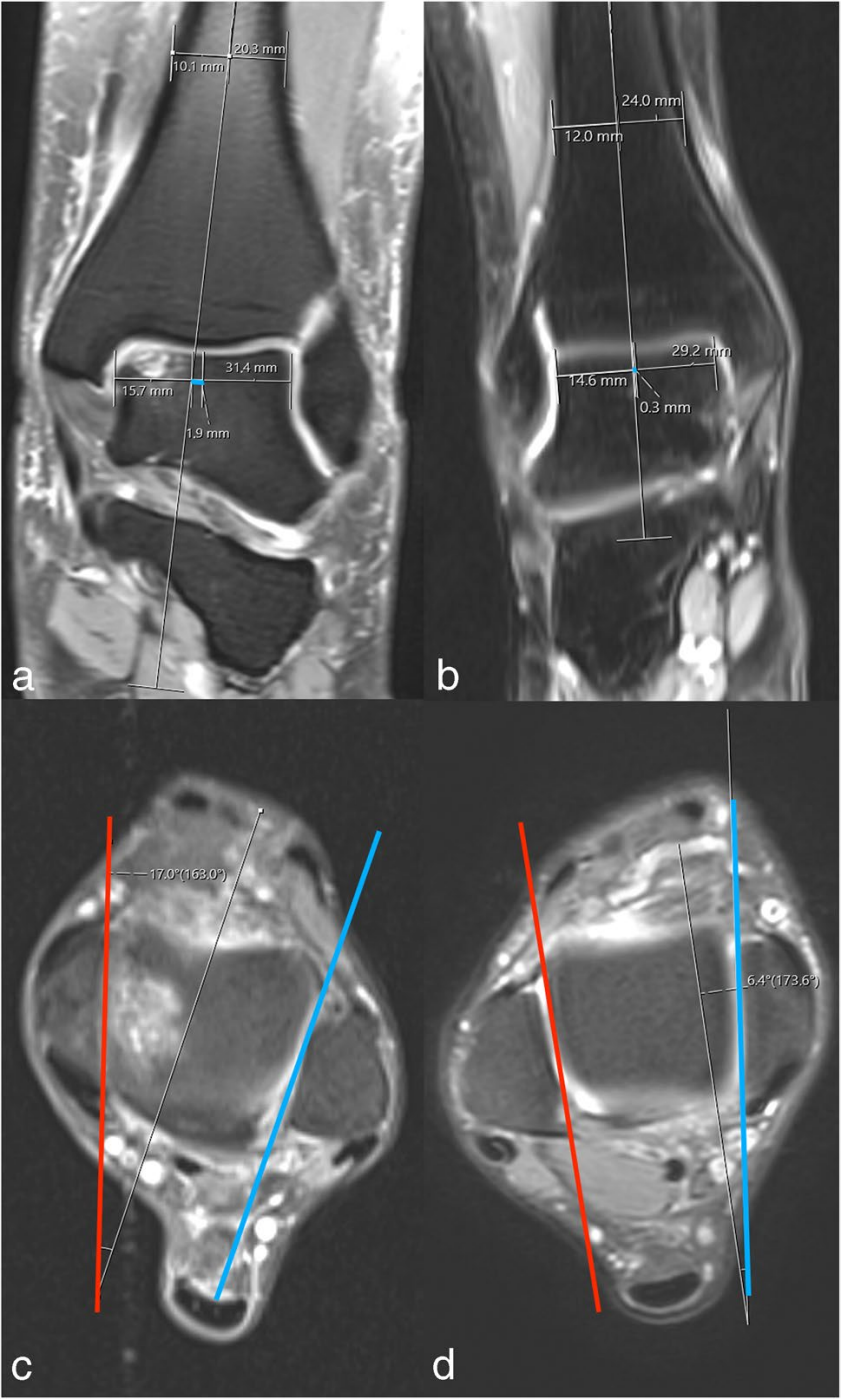
Measurements of talus position (TalPos) (a and b) and anterior opening angle of the talus (AOT) (c and d). In the osteochondral lesion case group, TalPos is more lateral (a) and AOT is larger (c) compared to the control group without an osteochondral lesion (b and d)

**Fig. 5:**
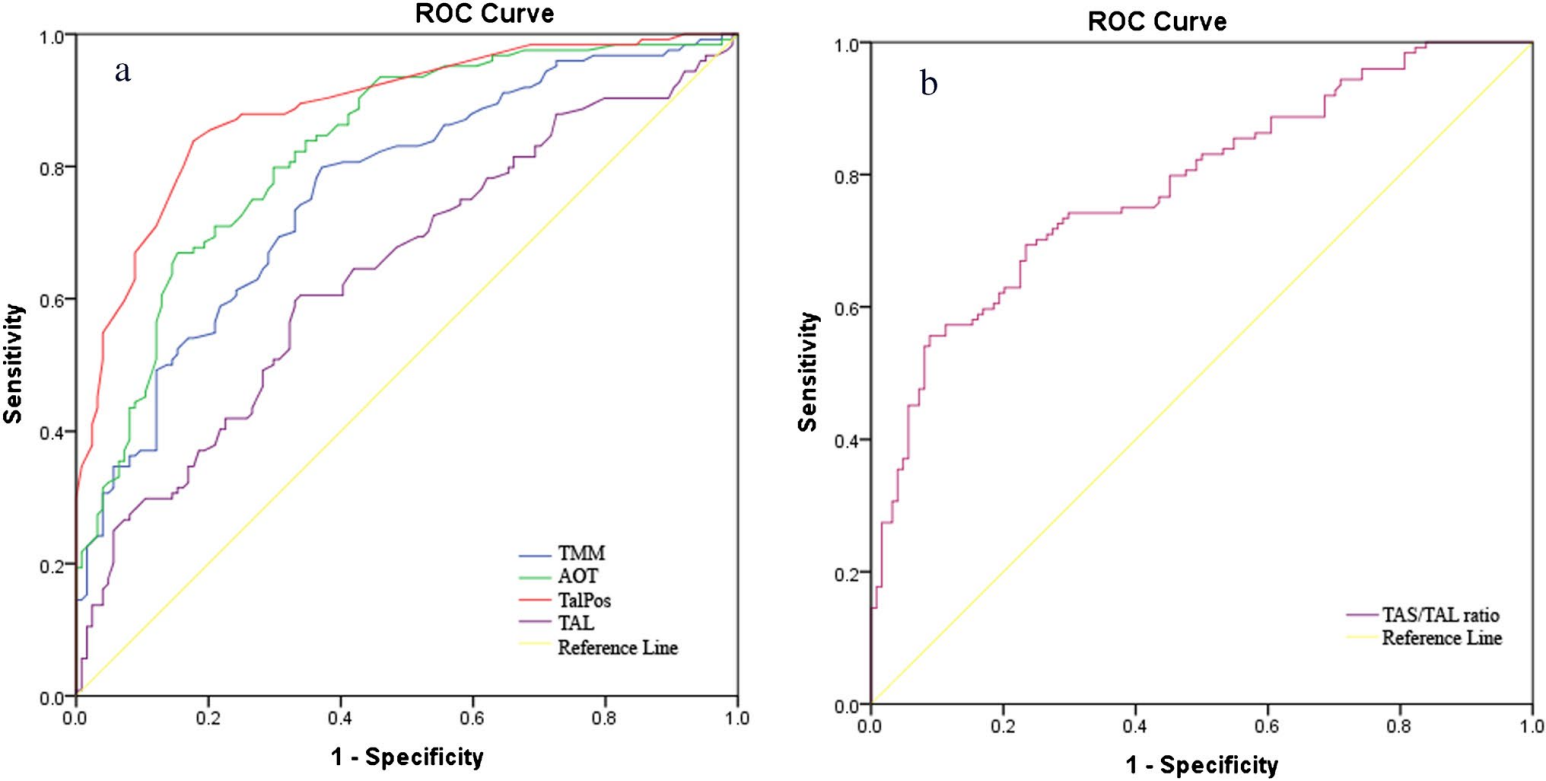
Receiver-operating characteristic (ROC) curves for the TMM (a), AOT (a), TalPos (a), TAL (a) and TAS/TAL Ratio (b) a. Larger results are more diagnostic for OLT; b. smaller results are more diagnostic for OLT

The TTM, AOT, TalPos, and TAL values were significantly higher in the case group than in the control group (*p* < 0.001). It was observed that the TAS/TAL ratio was statistically significantly lower in the case group than in the control group (*p* < 0.001). No significant difference was measured for TAS and IncDep (Table 3). Measurement differences are demonstrated as an example for the TalPos and AOT (Fig. 4). ROC analysis and AUC evaluated the discriminative power of TTM, AOT, TalPos, TAL, and TAS/TAL ratio, and cut-off values ​of these measurements were calculated and presented in Table 4. Cut-off value for TMM was 15.15 (sensitivity 69.4%, specificity 69.4%), for AOT 13.05 (sensitivity 66.9%, specificity 84.7%), for TalPos 0.75 (sensitivity 83.9%, specificity 82.3%), for TAL 35.45 (sensitivity 60.5%, specificity 66.1%), and TAS/TAL ratio 0.82 (sensitivity 69.4%, specificity 76.6%) (Fig. 5). Participants were categorized according to these cut-off values. Variables independently associated with OLT were evaluated with multivariate logistic regression analysis. OR = 5.1 for TMM ≥ 15.15°, OR = 10.2 for AOT ≥ 13.05°, OR = 39.6 for TalPos ≥ 0.75 mm, and OR = 7.1 for TAS/TAL Ratio ≤ 0.82. No association was observed between TAL and OLT (Table 5).

**Table 3:** Comparison of anatomical measurements between groups. Abbreviations, *SD* standard deviation, *TMM* tibial axis-medial malleolus angle, *AOT* anterior opening angle of the talus, *TalPos* talus position, *TAS* tibial articular surface, *TAL* length of trochlea tali arc, *IncDep* incisura depth

Anatomical parameters	Case group *n* = 124	Control group *n* = 124	*p* values
TMM mean (°) ± SD	17.6 ± 3.9	14.2 ± 2.7	< 0.001*
AOT median (°) (range)	14.3 (2–28.5)	8.7 (2–18.5)	< 0.001**
TalPos median mm (range)	1.6 (− 1.1 to 4.2)	0 (− 2.2 to 2.1)	< 0.001**
TAS mean mm ± SD	28.9 ± 3.9	29.4 ± 3.1	0.234*
TAL mean mm ± SD	36.8 ± 4.5	34.7 ± 3.6	< 0.001*
TAS/TAL mean ratio ± SD	0.79 ± 0.06	0.85 ± 0.05	< 0.001*
IncDep median mm (range)	4.35 (0–9.5)	4.4 (2–8.9)	0.219**

Bold entries are significant at <0.001

^*^Independent samples *T*-test; **Mann–Whitney *U* test

**Table 4:** Cut-off points, AUC values, sensitivity, and specificity of anatomical parameters as a predictive approach to OLT. Abbreviations, *OLT* osteochondral lesions of the talus, *AUC* area under the curve, + *LHR* positive likelihood ratio, *PPV* positive predictive value, *NPV* negative predictive value, *TMM* tibial axis-medial malleolus angle, *AOT* anterior opening angle of the talus, *TalPos* talus position, *TAS* tibial articular surface, *TAL* length of trochlea tali arc

Anatomical parameters	AUC (95% CI)	*P*	Cut-off	Sensitivity	Specificity	+ LHR	PPV	NPV	Max Youden Index
TMM	0.763 (0.705–0.822)	< 0.001	≥ 15.15	69.4%	69.4%	2.3	69.4%	69.4%	0.387
AOT	0.826 (0.775–0.877)	< 0.001	≥ 13.05	66.9%	84.7%	4.4	81.4%	71.9%	0.516
TalPos	0.887 (0.846–0.928)	< 0.001	≥ 0.75	83.9%	82.3%	4.7	82.5%	83.6%	0.661
TAL	0.642 (0.574–0.711)	< 0.001	≥ 35.45	60.5%	66.1%	1.8	64.1%	62.6%	0.266
TAS/TAL ratio	0.784 (0.728–0.841)	< 0.001	≤ 0.82	69.4%	76.6%	2.9	74.6%	70.9%	0.460

**Table 5:** Multivariate logistic regression analysis on independent association factors for OLT. Abbreviations, *OR* odds ratio, *TMM* tibial axis-medial malleolus angle, *AOT* anterior opening angle of the talus, *TalPos* talus position, *TAS* tibial articular surface, *TAL* length of trochlea tali arc

	Univariate logistic regression analysis		Multivariate logistic regression analysis model‡	
	OR (95% CI)	*p*	Adjusted OR (95% CI)	c_ontrolled*_
Age	1 (0.98–1.02)	0.435	0.97 (0.94–1.03)	0.079
Sex, female (ref: male)	1 (0.6–1.7)	1.000	1.7 (0.5–5.7)	0.374
TMM, ≥ 15.15° (ref: < 15.15°)	**5.1 (2.9–8.8)**	** < 0.001**	**5.9 (2.2–16.1)**	< 0.001*
AOT, ≥ 13.05° (ref: < 15.15°)	**11.2 (6.1–20.7)**	** < 0.001**	**10.2 (3.7–27.9)**	< 0.001*
TalPos, ≥ 0.75 mm (ref: < 0.75 mm)	**24.1 (12.4–46.8)**	** < 0.001**	**39.6 (14.1–111.4)**	< 0.001*
TAL, ≥ 35.45 mm (ref: < 35.45 mm) **	**2.9 (1.8–5.1)**	** < 0.001**	2.1 (0.6–6.6)	0.254*
TAS/TAL ratio, ≤ 0.82 (ref: > 82) **	**7.1 (4.1–12.5)**	** < 0.001**	**7.1 (2.8–17.9)**	< 0.001*

Bold entries are significant at <0.001

^‡^Cox & Snell R Square: 0.569

^*^Controlled for multiple testing using classic Bonferroni correction method (for 7 independent variables: family wise error rate = 0.3; *P*-value threshold = 0.05/7 = 0.007)

^**^Since the correlation (*r* =  − 0.289; *p* < 0.001) between TAL and TAS/TAL ratio was less than 0.3, two variables were included in the multivariate analysis as independent variables

## Discussion

The current study aimed to find morphological differences among subjects with or without OLT using ankle MRI. Four of five measured MR parameters differed significantly between the OLT case and control group in our study. The tibial axis-medial malleolus angle (TMM) and the anterior opening angle of the talus (AOT) were significantly larger in patients with an OLT. There was a shift of the talus towards the lateral side in the OLT group, and the TAS/TAL ratio was significantly smaller in favor of a greater TAL value in the OLT case group. TalPos and AOT had the greatest AUC values and odds ratios within the parameters, suggesting them to be the strongest predictive parameters. Thus, MRI morphological parameters of ankle morphology may be related to the occurrence of OLT.

There is some recent literature supporting our results. Masquijo et al. performed a similar MRI study and evaluated the relationship between OCD of the talus and morphometric parameters of the ankle in the pediatric-adolescent population. They could demonstrate a significant increase in the length of trochlea tali arc in ankles with juvenile OCD lesions compared with normal ankles [6]. A study in an adult population with medial OCD by Yurttas et al. showed a significantly larger malleolar width and length of trochlea tali arc for the OCD group compared with healthy volunteers [11].

Our study did not show a significant difference in the length of trochlea tali arc (TAL); however, the tibial coverage index was lower in the OLT patients, which means that the talar cartilage area covered by the tibia is relatively smaller. We assume this could lead to a higher pressure load on the talus and possibly reinforce OLT development. In our study, the anterior opening angle of the talus (AOT) was measured significantly greater for the OLT group compared to the controls. We assume the greater angle implies narrower posterior trochlea tali, which could be a possible destabilizing factor during plantarflexion. Especially, combined with weight loading, the reduced tibiotalar articulation surface could contribute to higher stress on the posteromedial aspect of the talus.

Another result of our study was the larger tibial axis-medial malleolus (TMM) angle in the OLT patients. Due to the lack of bony attachment between fibula and tibia, the bimalleolar retaining fork is rigid medially and movable laterally [21]. It is possible that a larger TMM angle provides more flexibility for the talus in-between the malleoli and thus may lead to destabilization in the ankle joint. This again may lead to an altered or focally increased weight load on the trochlea tali, which might be a co-factor for OLT development. A study by Teramoto et al. reported a complex evaluation of 3-dimensional CT data and could show that the medial malleolar articular surface and bony volume were smaller in patients with medial OLT than in a control group [12]. Similar to our results, they found larger anterior opening angles of the talus (AOT) and TMM angles in the OLT group than in the controls [12]. Another study supporting the importance of biomechanical factors on OLT etiology is an arthroscopic evaluation by Sugimoto et al. They identified a more significant talar tilt and a larger varus inclination of the tibial plafond to be associated with high-grade chondral lesions and to accelerate chondral degeneration [15]. A notable consideration of their study was that mechanical instability of the ankle and inclination of the tibial plafond are surgically correctable [15].

Other studies described not only the tibial plafond angle but also the position of the fibula in the horizontal plane as being important morphological parameters in patients with ankle instability that may predispose to OLT [22, 23]. However, it is challenging to measure the position of the fibula by means of MR images, and especially the dynamic behavior of the lateral malleolus during weight load remains non-registered. Our initial assumption that the depth of the incisura fibularis could be representative of the fibular stability was refuted; there were no significant differences between the OLT and control groups. The syndesmosis and ligaments probably play a decisive role in fibular positioning and can compensate for many osseous conditions, such as a shallow configuration of the incisura fibularis.

Another important result of our study was the significant difference of the talar position within the bimalleolar fork towards the lateral side in the OLT patient group. We attribute the change of the talar position as a possible disruptor of joint congruence and, therefore, a disadvantageous change in the tibiotalar contact area and weight distribution on the talar cartilage. An anatomical study of tibiotalar articulation by Ramsey and Hamilton revealed the greatest reduction in the contact area between the tibia and talus occurring during the initial 1 mm of lateral displacement of the talus, with the average reduction being 42% [16]. Similarly, the tibiotalar contact area decreased about 45% when the distal fibula was replaced 2 mm laterally [24].

Our study has some limitations. There was no matching of the control group for height, weight, and lower limb alignment, which may be possible confounders. The control group did not consist of entirely healthy individuals but mainly included patients with unspecific ankle pain, potentially implying an OLT in the earliest stage. Despite a high level of standardization, very precise MRI measurements are difficult to reproduce, e.g., due to varying ankle positioning during the examination or osteophytes, which could impede the assessment of the joint surface.

In general, the combination of mechanical properties, the vascular vulnerability of osteochondral areas, and the genetic susceptibility of some individuals leave the etiology of OLT still multifactorial. In our study, OLT patients showed significantly different morphological parameters of the talus and the distal tibia. TMM, AOT, and CI are fixed anatomical measurements; hence, we assume they could be congenital predisposing factors for the development of OLT. TalPos and IncDepth are connected to the fibula position and, therefore, might be affected by the stability of the syndesmosis and ligamentous structures. Thus, there is a possibility that these parameters could be acquired during a lifetime by functional loading, e.g., sports activities. Moreover, they might be fewer contributing factors, but more the effect of an OLT, e.g., due to pain-associated false weight loading. An important strength of our study is that all parameters are independent of patient size as they were angle measurements, or a normalization was performed by index calculation. We think a standardized approach is essential, e.g., as Kuo et al. could even show that some morphological ankle parameters significantly differed between Chinese and Caucasian subject groups [13].

In a clinical setting, the major goal remains the detection of possible contributing factors of OLT, and the evaluation if their correction is a treatment option for the particular individual. Future studies could evaluate if there is a correlation between ankle morphology and OLT stage and progression or clinical symptoms such as pain or impaired mobility. Regarding OLT treatment, it would be necessary to evaluate if the correction of anatomical parameters could positively influence OLT development and reduce the progression.

In conclusion, our study supports the assumption that ankle morphology might have an influence on OLT development. Especially, the talus position and the anterior opening angle of the talus (AOT) are important associated parameters and seemed to be the strongest predictive factors for OLT development. However, due to a multifactorial process, the etiology remains unclear. Further studies are necessary to evaluate the predisposing factors of OLT in more detail and determine the best treatment options for OLT patients.

## Abbreviations

AOT: Anterior opening angle of the talus
AUC: Area under the curve
CI: Confidence interval
+ LHR: Positive likelihood ratio
MRI: Magnetic resonance imaging
NPV: Negative predictive value
OLT: Osteochondral lesion of the talus
OD: Osteochondritis dissecans
OR: Odds ratio
PACS: Picture archiving and communication system
PDw: Proton density-weighted
PPV: Positive predictive value
ROC: Receiver-operating characteristic
SPIR: Spectral presaturation inversion recovery
TAL: Length of trochlea tali arc
TalPos: Talus position
TAS: Length of the distal tibial articular surface
TMM: Tibial axis-medial malleolus angle
TSE: Turbo spin-echo
